# Artificial cells for in vivo biomedical applications through red blood cell biomimicry

**DOI:** 10.1038/s41467-024-46732-8

**Published:** 2024-03-20

**Authors:** Jorik Waeterschoot, Willemien Gosselé, Špela Lemež, Xavier Casadevall i Solvas

**Affiliations:** Department of Biosystems - MeBioS, KU Leuven, Willem de Croylaan 42, 3001 Leuven, Belgium

**Keywords:** Biomimetic synthesis, Drug delivery, Biosensors

## Abstract

Recent research in artificial cell production holds promise for the development of delivery agents with therapeutic effects akin to real cells. To succeed in these applications, these systems need to survive the circulatory conditions. In this review we present strategies that, inspired by the endurance of red blood cells, have enhanced the viability of large, cell-like vehicles for in vivo therapeutic use, particularly focusing on giant unilamellar vesicles. Insights from red blood cells can guide modifications that could transform these platforms into advanced drug delivery vehicles, showcasing biomimicry’s potential in shaping the future of therapeutic applications.

## Abstract (web summary)

Despite the enormous progress in the field of giant lipid vesicles, their use for in vivo biomedical applications is limited. Here, the authors discuss red blood cells as inspiration for enhancing those vesicles, investigating the required cellular features and the corresponding technical hurdles.

## Introduction

Artificial cells are cell-like compartments that are able to reproduce at least one of the basic properties of cells: ability to compartmentalise enzymatic reactions, store and replicate information, exchange mass and energy with their environment, self-organise and regulate spatiotemporal features, sense and communicate with their environment, move, grow, reproduce and evolve^1^. Although the compartmentalisation property has been demonstrated in many different systems (i.e., membrane-based - such as in liposomes and polymersomes - or membrane-less - such as in emulsions, colloidosomes and coacervates)^2^, in order to replicate the functions of cells that occur at their surface (i.e., communication, division) the most promising approach is to build a compartment consisting of a lipid membrane hosting functional membrane proteins. Towards this end, the use of lipid vesicles consisting of a single lipid bilayer represents the most straightforward route, and, in particular, Giant Unilamellar Vesicles (GUVs) are ideal candidates since their dimensions (i.e., with diameters above 1 μm) and curvature are the most similar to eukaryotic cells^3–5^. This type of lipid vesicles has been proposed for use in a wide variety of applications, ranging from the study of cell division^6^, to insulin-producing artificial cells^7^. Despite their attractive features, these biomimetic membrane systems have become stagnant as a model system for fundamental research, without progressing into any major translational applications and virtually no clinical trials published on their potential for therapeutic use. This is in striking contrast with their smaller counterparts, Large Unilamellar Vesicles (LUVs), with diameters between 100 nm and 1 μm (commonly know as liposomes), which have been used in countless clinical applications^8–10^. Although at first glance this might appear rather surprising, given the similarities between GUVs and eukaryotic cells which routinely perform a wide set of therapeutic functions in vivo, these artificial systems are typically not stable enough to remain in circulation for long enough to provide an effective therapeutic value.

In this review we highlight the efforts made so far to produce therapeutic GUVs, taking on a biomimetic approach to shed light on what are the requirements for GUVs to become successful therapeutic artificial cells for in vivo applications. Specifically, we focus on the biomimicry of human red blood cells (RBCs), as they are a relatively simple cell model that can remain in circulation for up to 120 days^11^. We discuss the key aspects that provide RBCs with such exceptional biocompatibility (like size, shape, lipid composition, osmotic balance and macromolecular crowding) and we examine how these features can be mimicked within a GUV platform. Additionally, we review and compare, several RBC-mimicking carriers based on different systems, including GUVs, LUVs and modified RBCs. Finally, we list several important properties beyond advancements in RBC biomimicry that are needed to drive the nascent field of therapeutic artificial cells forward.

## GUVs and RBCs: the basics

### GUVs

The study and utilisation of GUVs has traditionally been constrained to obtaining fundamental insights in lipid and protein-lipid mechanics (*e.g*., actin-driven tubulation^12^, vesicle fusion^13^ and vesicle budding^14^). The scientific community, though, quickly realised that these compartments could also be proposed as cell-like environments with immense potential for research on the origins of life as well as diverse biomedical applications^4,15^. Just like the smaller liposomes, GUVs can be produced with well-defined materials, synthesis and purification processes. They can be further functionalised with a diversity of biomolecules, fuse with cell membranes for intracellular drug delivery and vehiculate both hydrophilic and hydrophobic molecules^16–18^. In regards to the latter, GUVs offer some inherent advantages: due to their large size they can encapsulate large quantities of drugs and also accommodate the transport of larger drug constructs^19,20^, which opens up the possibility of controlled release over long periods of time to maintain optimal drug levels^6^. GUVs can also contain multiple subcompartments, enabling the integration of complex signalling-like pathways where biochemical signals can trigger specific responses (i.e. drug release). Finally, due to their size, they can also modulate and mimic cellular processes, as shown in investigations of immune cell interactions^21^.

#### GUV production: state of the art and limitations

In order to fulfil the previous applications, it is crucial to use adequate methods to produce GUVs. And although a wide set of techniques exists for GUV generation^22^, these typically suffer from limitations in the structure and composition of the GUVs they can produce. In general, GUV production methods can be divided in three categories: (1) the lipid film hydration methods, (2) the inverted emulsion transfer methods, and (3) the microfluidic methods. The latter can be further subdivided into the double emulsion methods and the surfactant-mediated methods. Below, these methods are briefly introduced with a special attention highlighting some of the current limitations concerning the complex production of GUVs for biomedical applications. For a more in-depth overview on the different techniques for GUV production, the reader is invited to examine two excellent recent reviews^22,23^.

##### Lipid film hydration method

**Fig. 1:**
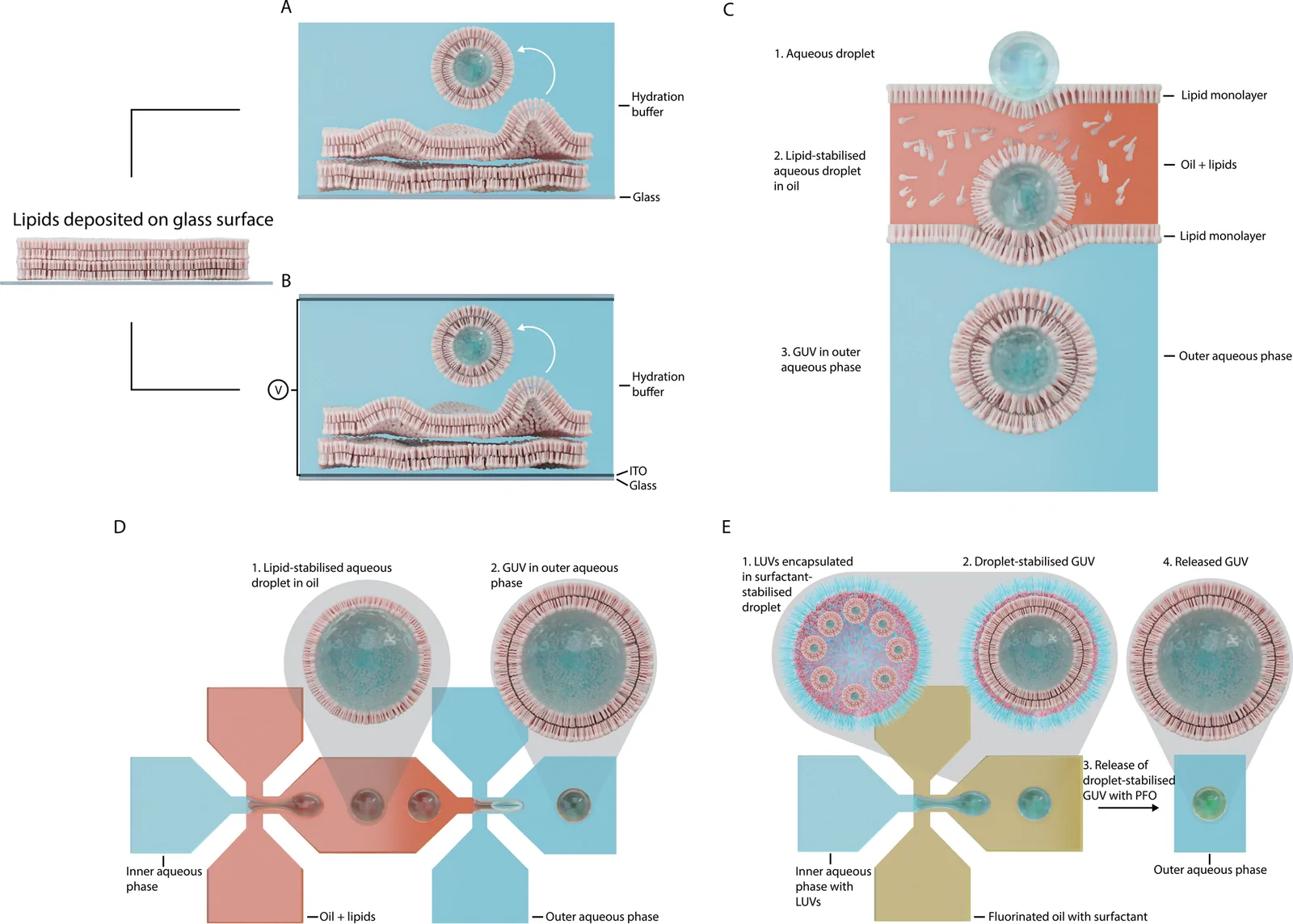
Illustration of five different methods for GUV creation. **A** Thin film hydration. **B** Electroformation. **C** Phase transfer method. **D** Microfluidic double emulsion method. **E** Microfluidic surfactant-based method.

In this technique, illustrated in Fig. 1A, a dehydrated lipid film is deposited on a solid substrate, *e.g*., the bottom of a round bottom flask. The film is then hydrated with an aqueous solution after which the lipid film undergoes a swelling process resulting in GUV formation^24^. This method, though, is highly limited in the buffer and lipid conditions that can be used and often results in the production of multilamellar vesicles^25^. Therefore, many variants around this method have been developed, the most common incorporating an electroformation step wherein the lipid film is deposited on a conductive surface (like indium tin oxide-coated glass or platinum wires) and subjected to an AC voltage which effectively speeds up the lipid swelling into GUVs, as depicted in Fig. 1B^26^. Although this strategy affords a more homogeneous GUV dispersion with higher yields, it is restricted to the use of lipids with neutral net charge and buffers with low ionic strength, which hinders the possibility of working with physiological buffers and the integration of a wide set of functional proteins. Furthermore, the encapsulation efficiency of reagents inside the GUVs is rather low^26^. Another variation is performed by drying and hydrating the lipids on top of a polymer-based hydrogel, where the lipid swelling is enhanced by influx of hydrating solution from below. Different hydrogels have been used, including agarose, polyvinyl alcohol, and dextran polymers, among others. This method allows the use of a wider set of buffer and lipid conditions, but the obtained vesicles remain polydisperse^3^.

##### Inverted emulsion transfer method (IET)

Unlike thin film hydration methods, IET methods allow efficient encapsulation of reagents and enable a better control on the size and lamellarity of the produced GUVs^27^. This technique exploits the spontaneous self-assembly process of amphiphilic lipid molecules into a monolayer at the interface that results from generating a water-in-oil (w/o) emulsion. When the aqueous droplets in this emulsion are forced to move across an additional w/o interface harbouring another lipid monolayer, this second monolayer wraps around the first one forming a lipid bilayer, which results in the formation of a GUV (Fig. 1C). This method accommodates the generation of GUVs with asymmetric bilayers in which biological compounds can readily be encapsulated^28^. Although effective for simpler applications, this strategy is also heavily burdened by several limitations: a major issue is reproducibility, which is highly dependent on variations in the composition of the oils used and environmental conditions^23,29^. Furthermore, the exact lipid composition of the GUVs is difficult to tune, due to differences in adsorption kinetics of the lipid species at the w/o interface. Also, traces of oil tend to remain within the final lipid bilayer, which deteriorate its properties and curtail the function of integrated membrane proteins. Additionally, these membrane proteins can only be added afterwards by spontaneous insertion, by fusion with protein containing liposomes or they have to be produced inside the GUV via an in vitro translation system, which highly limits the repertoire of membrane proteins that can be inserted in the produced GUVs. Optimisations of this method include performing the lipid preparation in a humidity-controlled glove box and using microfluidic components^5,30^ or microcapillaries^31^.

##### Microfluidic methods

Similar to the previous method, in this strategy a water-in-oil-in-water (w/o/w) double emulsion is typically generated in a microfluidic chip, with each interface being stabilised by a lipid monolayer. The oil layer is then removed either by extraction of the solvent^32–34^ or through interfacial forces^35^, to finally deliver a GUV, as illustrated in Fig. 1D. Again, full removal of the oil from the bilayer is difficult^36,37^, so this method suffers from similar limitations as the IET method.

To bypass these restrictions, Weiss et al.^38^ developed a method that does not require any transfers through a potentially contaminating oil. In this strategy LUVs or SUVs (small unilamellar vesicles, with diameters under 100 nm) are encapsulated in w/o droplets stabilised by surfactants. By tuning the interaction strength between the surfactants and the lipids of the encapsulated vesicles, the latter can be induced to fuse at the w/o interface to form a droplet-stabilised GUV (dsGUV), which then needs to be released from the oil environment into an aqueous environment (Fig. 1E). Although the preparation of such w/o droplets does not intrinsically require a microfluidic platform (i.e., any emulsification process can be applied^39^), complex microfluidic techniques (*e.g*., picoinjection) are essential for the introduction of certain biomolecules (*e.g*., actin filaments) into the GUVs. Additionally, the lipid composition and buffer conditions have to be carefully chosen to efficiently release the GUVs from the droplet environment^40^, a procedure which, in itself, is not trivial to perform and requires the use of a demulsification reagent that may negatively affect the function of any inserted membrane proteins. Recently, these issues have been addressed by replacing the surfactant with nanoparticles^41^, which provides additional benefits, including a better compartmentalisation of the encapsulated biomolecules and the ability to integrate complex transmembrane proteins in the GUVs.

Table 1 provides an overview of the advantages and disadvantages of all these methods. In conclusion, in spite of a wide diversity of techniques available for GUV production, these strategies are generally ill-suited for manufacturing complex, biologically relevant GUVs featuring cell-like properties (i.e., lipid asymmetry, presence of lipid-raft-like nanodomains and complex membrane proteins and protein complexes), which is necessary to enable significant advances in the generation of artificial cells for biomedical applications. Nevertheless, to bestow advanced functionality to GUVs (i.e. targeted delivery, sensing capacity, stability, ...), many of these production techniques have been expanded or modified to accommodate the reconstitution of specific transmembrane proteins in the GUVs’ lipid bilayer, which the reader is invited to peruse in the excellent recent review from Litschel et al.^22^. Finally, although these functionalised GUVs have already been applied in vitro, they have only been used in vivo in exceptionally rare occasions. In the following sections these most advanced applications are reviewed in detail.

**Table 1:** Comparison of different GUV production methods

	Film hydration	Emulsion transfer	Microfluidic (double emulsion)	Microfluidic (surfactant-mediated)
Ease of use	+++	++	+	+
Encapsulation efficiency	+	+++	+++	+++
Monodispersity	+	++	+++	+++
Unilamellarity	+	+++	+++	+++
Lipid and buffer variety	+	++	++	++
Production throughput	+	++	+++	++
Membrane protein incorporation	+	+	+	++
Oil Contamination	No	Yes	Yes	No
Leaflet asymmetry	No	Yes	Yes	No
Comment		Process variabilities		

#### In vitro examples

In 2022, Hernandez et al.^42^ demonstrated the feasibility of using GUVs to target and induce apoptosis in Jurkat cells by functionalising the outer GUV surface with an antibody complex for cell-recognition and an apoptosis-inducing protein (FasL ligand), providing a first proof of concept of a cytotoxic synthetic cell.

**Fig. 2:**
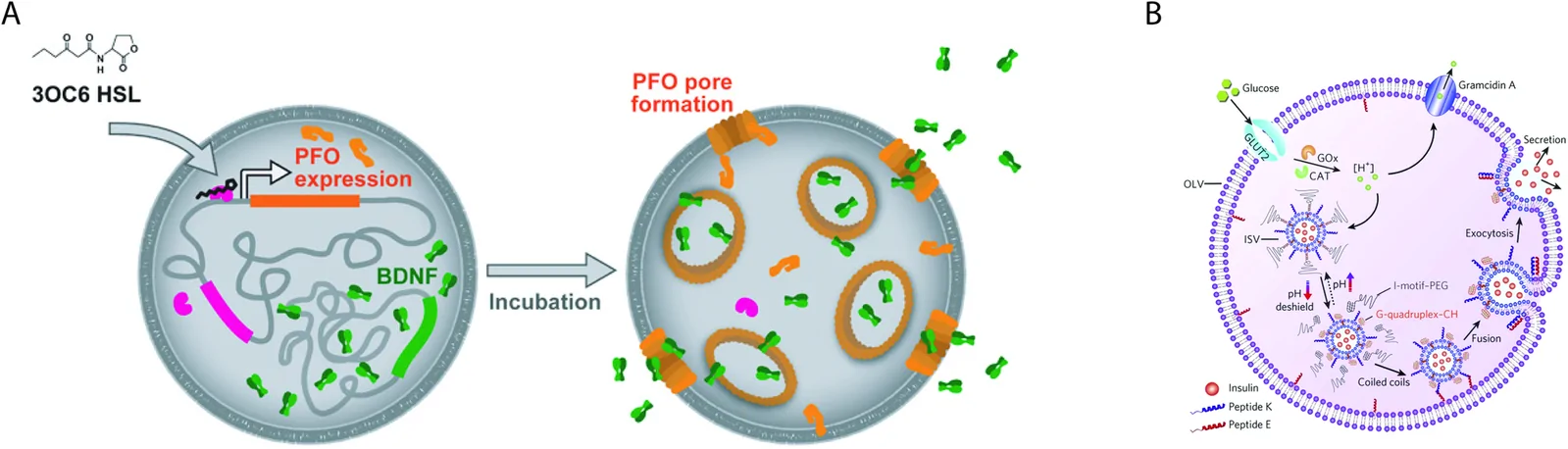
Two examples of potential artificial cell applications. **A** Neurotrophic factor-producing artificial cells (used under CC-BY license)^43^. **B** Artificial beta cells (reprinted with permission)^7^.

In another work, Toparlak et al.^43^ developed GUVs encapsulating a responsive in vitro transcription/translation system capable of producing a neurotrophic factor in combination with a pore-forming protein enabling the release of the factor into the outside medium (see Fig. 2). The translation of these proteins was triggered in respons to the presence of lactones allowing for target specificity. Notably, the GUV solution had to be exchanged every 24 h for optimal performance.

A final, relevant example, was developed by Staufer et al.^44^, who created GUVs acting as artificial cellular organelles. This work encompassed the fabrication of synthetic peroxisomes with bovine catalase supporting cellular stress management, synthetic endoplasmatic reticulum acting as light-responsive calcium storage and magnetosomes encapsulating iron nanoparticles. The synthetic organelles exhibited survival up to 72 h when incubated with HaCaT and NRK cells before being degraded.

#### In vivo examples

While in vitro studies have demonstrated the therapeutic potential of GUVs, research on their application in vivo is still very limited, given their fragility.

In a first interesting study by Chen et al.^45^, GUVs that produced recombinant human basic fibroblast growth factor were incubated with endothelial cells and, later on, locally administered in mice through implanted matrigel plugs. These GUVs showed pro-angiogenic activity both in vitro and in vivo and were demonstrated to be non-immunogenic, albeit they were only active for 6 h.

In a second paradigmatic exampled, Krinsky et al.^46^ evaluated the use of GUVs excreting *Pseudomonas* exotoxin for the treatment of 4T1 breast cancer. First, these GUVs were incubated in vitro with 4T1 breast cancer cells, upon which 80% of the cancer cell population was killed. Next, these GUVs were tested in vivo via injection into tumours induced in the mammary fat pad of BALB/c mice, resulting in an increase of the expression of caspase 3, an apoptotic marker.

In an earlier study, Chen et al.^7^ produced GUVs capable of releasing insulin at high glucose concentrations as a demonstrator of artificial beta cells. GUVs were formed encapsulating glucose oxidase and peptide-shielded LUVs which encapsulated insulin. Upon the intake of glucose, this was oxidised, resulting in a decrease in pH inside the GUVs which, in turn, sterically deshielded the peptides tethered to insulin-loaded LUVs. As a result, this first set of peptides could associate with a second set of peptides, anchored to the inner surface of the GUV, inducing the fusion of the LUVs with the GUV membrane and the release of the encapsulated insulin (Fig. 2). These GUVs were dispersed in a hydrogel-forming solution which was subcutaneously injected in streptozotocin-induced type 1 diabetic mice. With this system, glucose levels could be kept normal for up to five days in the treated mice.

These compelling examples highlight both the therapeutic potential of artificial cells based on GUVs as well as the fact that the long-term in vivo survival of these systems is one of the major hurdles that needs to be overcome for their widespread application. With respect to this limitation, in the following chapters we review how RBC mimicry can be used to further improve the longevity of GUVs in in vivo applications.

### RBCs

**Fig. 3:**
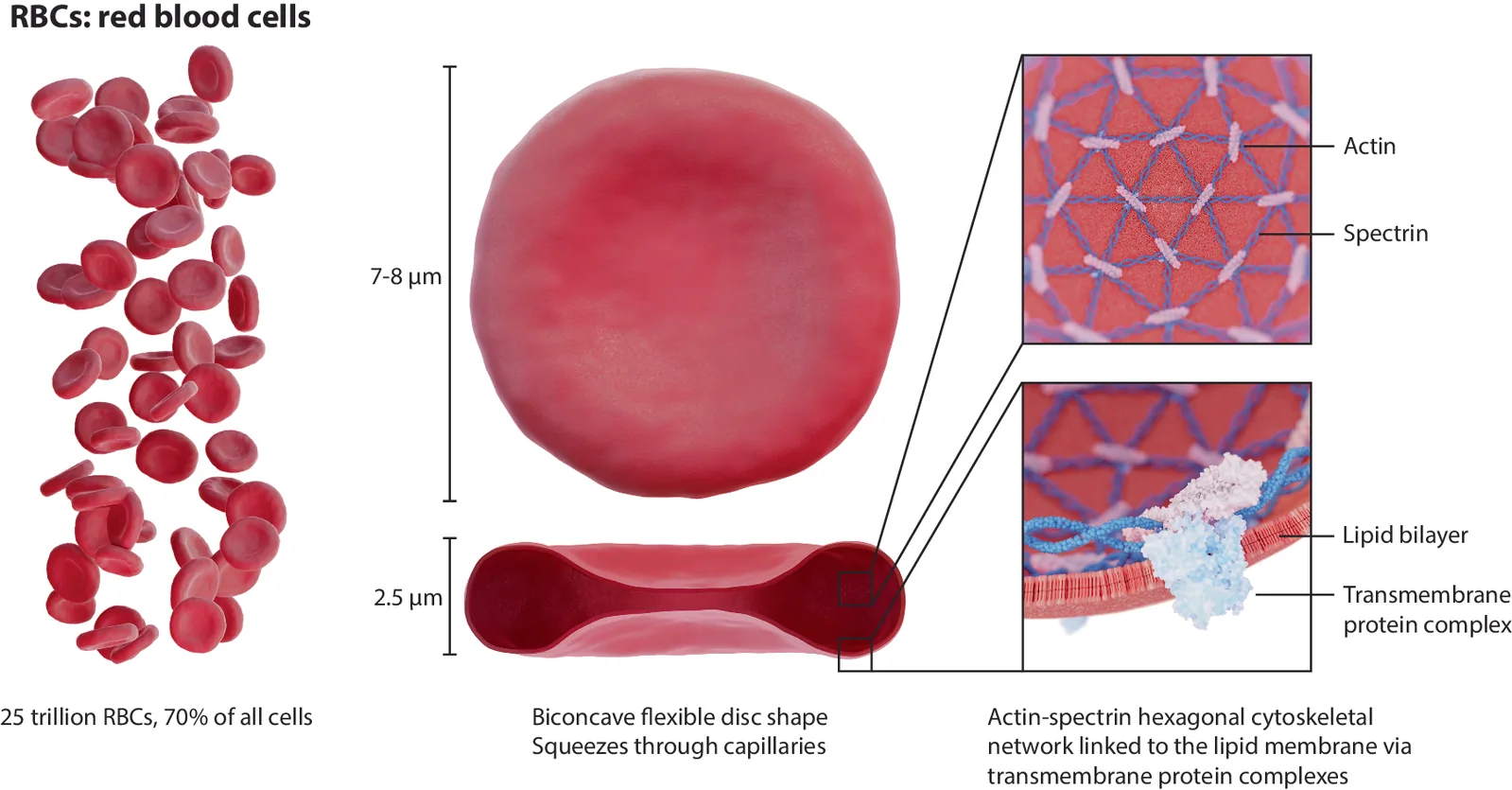
RBC properties. RBCs are the most abundant cell type in the human body. They have a biconcave disc shape and are extremely flexible, enabling their passage through capillaries and slits many times smaller than themselves. This flexibility is a result of their highly specialised cytoskeleton, composed of an actin-spectrin cortex (top right) that is linked to the lipid bilayer by membrane-associated protein complexes (bottom right).

RBCs are the most abundant cell type in the human body and typically circulate for 90–120 days before they are cleared in the spleen^11^. The long-term stability of RBCs is a result of a complex interplay between their morphology, membrane composition and mechanical properties, which allow RBCs to squeeze through the smallest conduits of the capillary beds, enabling them to access every tissue in the human body (Fig. 3 for a visual overview). Additionally, RBCs possess a wide set of transmembrane proteins and glycoproteins which mediate their interaction with and prevent their removal by the immune system^16^. For these reasons, it has long been proposed that the biomimicry of RBCs can be used as a strategy to engineer drug-carrying vehicles affording long-term drug bioavailability and responsive delivery, which would greatly improve the pharmacokinetics of a wide diversity of therapeutic agents (and, particularly, large biologicals)^47,48^, while, at the same time, allowing to target all organs, tissues and cell types. Furthermore, RBCs interact with the immune cells in the blood, liver and spleen, which also provides a route for developing immunotherapies based on synthetic antigen-presenting cells (APCs)^49^. Additionally, since RBCs are cleared in the spleen by phagocytes, a biomimetic RBC variant would allow for a simple strategy to specifically target these cells^47^. Finally, being arguably the ‘simplest’ type of eukaryotic cells (i.e. lacking organelles and nucleus), RBCs are an ideal model to base the development of biomimetic artificial cells for therapeutic purposes^11^.

## RBCs as biomimetic systems

**Fig. 4:**
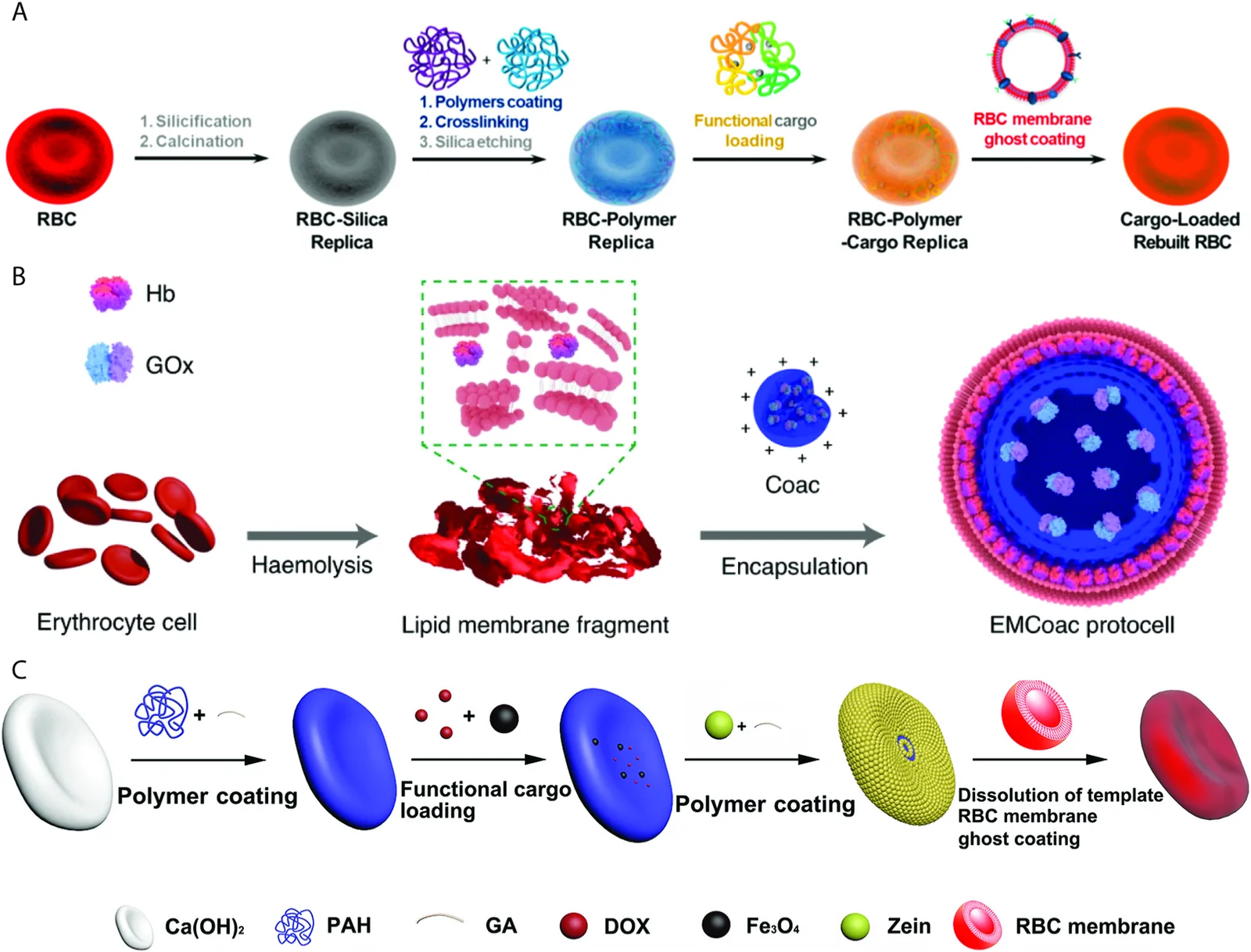
RBC biomimetic systems. **A** Polymer-RBC replica produced using RBCs as a template (reprinted with permission from ref. ^51^ Copyright 2020, American Chemical Society). **B** Coacervate coated with RBC membrane fragments (reprinted with permission from ref. ^52^). **C** Mimic produced by coating calciumhydroxide particles with first a polymer solution, then zein particles and finally RBC membranes are utilised to cover up the particles (reprinted with permission from ref. ^53^ Copyright 2022, American Chemical Society).

Mimicking the properties of RBCs for enhanced therapeutic vehicles has been an enduring idea through the years^48^. Still, most of the strategies that have been pursued to date (see “RBC-based systems” section) do not involve the manufacture of GUVs with RBC-like properties. Here, three such examples are presented which we found to be paradigmatic for their complexity and for the inclusion of more than one RBC property simultaneously (Fig. 4). More examples of RBC mimics can be found in review^50^, however, little in vivo applications have been reported to date.

The first relevant example of RBC-like GUVs was reported by Guo et al.^51^, who produced an RBC mimic by first replicating the RBC shape via a silification/calcination process followed by a layer-by-layer deposition of a polymer. After this deposition, the silica template was etched away and the resulting polymeric structures were coated with an RBC membrane. These vesicles were shown to mimic the size, biconcave shape, outer membrane and oxygen-carrying capability of RBCs. What is more, they could even be loaded with drugs (doxorubicin), magnetic nanoparticles and a toxin biosensing system (based on ATP sensing). These RBC mimics were then tested *ex ovo* in chick embryos and in vivo in mice, whereby it was shown that both the system’s flexibility and membrane coating were essential for survival in the bloodstream. Despite the emulation of many of the RBCs properties, the elimination half-life was calculated to be 41.8 h, which is still vastly smaller than that of actual RBCs.

In an attempt to mimic the longevity and the circulatory properties of RBCs, Liu et al.^52^ prepared GUVs by coating dextran/dsDNA coacervate droplets with haemoglobin-containing RBC fragments. In addition, the droplets co-encapsulated glucose oxidase, which, once triggered by the presence of glucose and hydroxyurea, enabled the production of nitric oxide (NO). The RBC mimics were injected intravenously into rabbits where the produced levels of NO were capable of stimulating blood vessel vasodilation, albeit only 25% of these mimics survived for 2 h and practically all of them were cleared within 24 h.

In the last example, Hou et al.^53^ demonstrated a method to shape zein (a maize protein) particles in the form of RBCs which were then coated with RBC membrane fragments^53^. These RBC mimics were incubated with the anti-cancer drug doxorubicin and Fe_2_O_3_ nanoparticles for in vivo imaging. Upon injection in mice, these particles had a retention rate of 28% after 24 h.

**Fig. 5:**
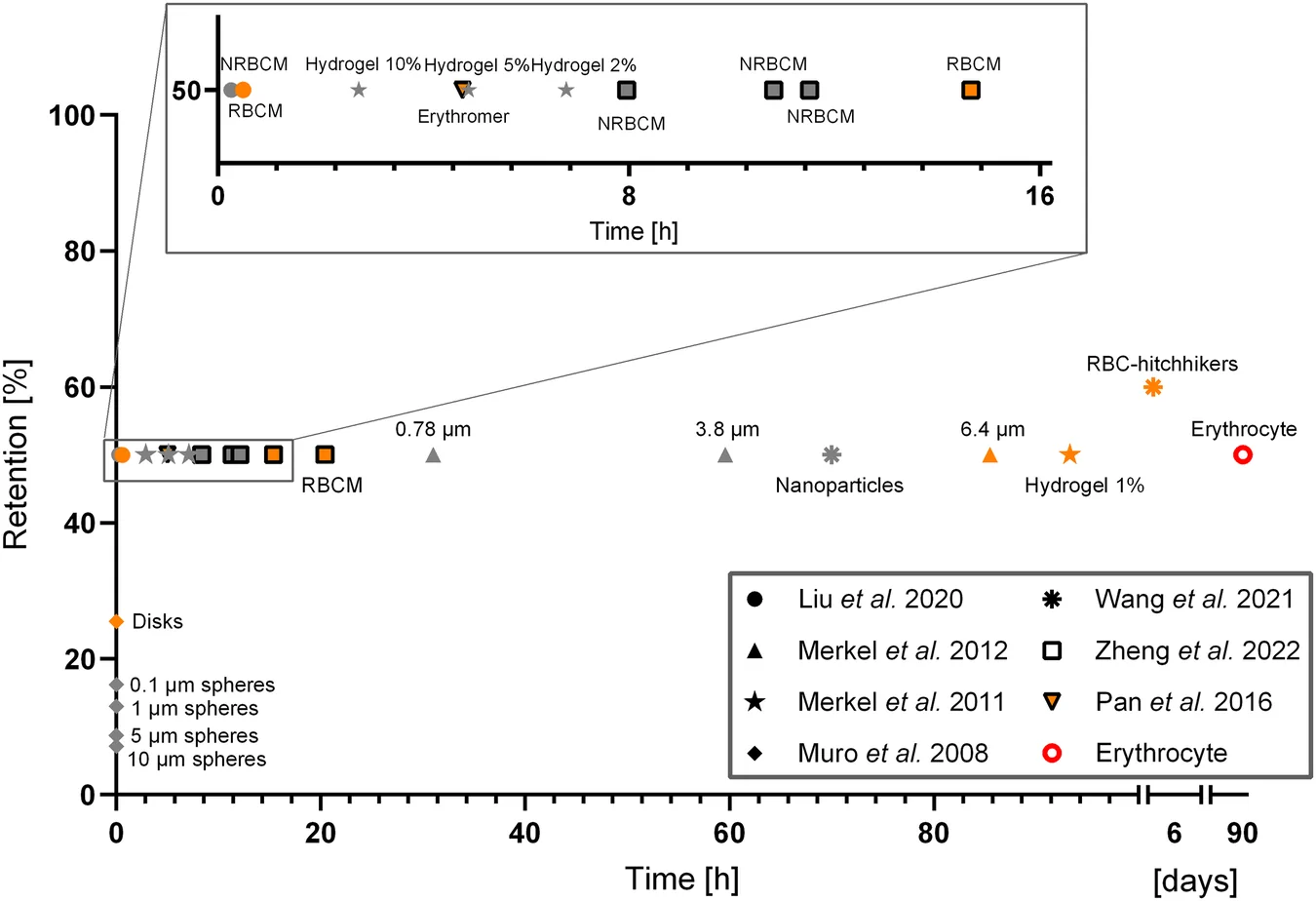
The graph above summarises some of the studies cited in this review, highlighting in vivo retention in the circulatory system as a function of time. Each symbol corresponds to a specific paper, the orange colour is for samples that more closely mimic RBCs. RBCM stands for RBC membrane-coated samples in contrast to NRBCM, which are the free non-RBC membrane-coated particles. While these studies are not fully comparable due to differences in materials, size and shape of the particles tested, as well as the studied species, all studies show a clear trend of prolonged retention when the vehicles integrate some of the RBC-related properties. As seen from the graph, the particles developed by Muro et al. present improved retention when produced with a discoid shape, as compared to their spherical counterparts. In the study of Merkel et al., lower percentage of cross-linker in the hydrogel, which contributed to a higher flexibility of the particles, improved retention. Merkel et al. showed that discoid-shaped hydrogel particles had an improved circulatory retention when the size of the particles resembled the size of RBCs. In Liu et al., Wang et al., & Zheng et al., an increased retention of RBC membrane-coated particles in comparison to their uncoated counterparts was demonstrated^52,62–64,161,162,194^.

These three studies highlight the enormous potential of harnessing an RBC biomimetic approach to develop a next generation of drug-delivery devices, although they also stress the intrinsic limitations that these approaches present with regards to biocompatibility. To improve on this facet, it is essential that these biomimetic carriers adequately reproduce the physicochemical properties of RBCs, properties that are outlined below. Additionally a set of different (non)-biomimetic examples are compared in Fig. 5, providing additional indication for the potential of RBC biomimicry.

### Morphology and mechanics

RBCs can be regarded as the ‘contortionists’ of the mammalian cell repertoire. To be able to squeeze through narrower cavities than their own dimensions (such as blood capillaries and the spleen’s interendothelial slits), their distinctive biconcave shape is temporarily lost to adopt a diversity of particular shapes that depend on the degree of shear to which the RBCs are exposed^54–56^. This phenomenon is directly linked to their morphology (size and shape) and their membrane mechanics, which are both largely determined by their cytoskeleton.

#### Size and shape

Lacking a nucleus, organelles and extensive cytoplasmic structures (modifications that occur during erythropoiesis), mammalian RBCs have also evolved to adopt a biconcave shape, which is dictated by their highly specialised cytoskeleton, surface area and cellular volume^57,58^. With a 40% additional surface area compared to spherical cells with the same volume, RBCs are highly deformable (see “Mechanics and deformability” section>), and have optimised internal diffusion processes by eliminating the dead volume that spherical cells have at their core^59^. With a diameter of 7–8 μm and a height of 2.5 μm, RBCs are one of the smallest mammalian cell types. Pathological RBC morphologies present altered shapes and/or surface area-to-volume ratios (*e.g*. as in hereditary spherocytosis), all of which are subjected to increased retention rates in the spleen^60,61^. Merkel et al.^62^ evaluated the in vivo circulation of RBC-shaped hydrogel microparticles of several sizes in mice, demonstrating that particles with the native RBC diameter (~6.4 μm) stayed in circulation the longest (Fig. 5). Smaller particles were cleared quickly by the immune system, while larger particles became physically trapped in the liver and spleen, rapidly falling out of circulation. Similarly, this and other studies showed that microparticles with a biconcave or discoidal shape have a significantly longer circulation half-life than their spherical counterparts^63,64^. The exact mechanisms behind this remain unclear, but are hypothesised to be related to the altered flow mechanics, organ-specific preferences for certain particle shapes and improved flexibility in at least one dimension^65,66^.

The biconcave RBC shape has been replicated in many different processes and substrates. Via electrospraying, biconcave microparticles of, for example, chitosan/terephthaldehyde, pectin/oligochitosan and ethyl(hydroxyethyl)cellulose have been produced^67–69^. Other groups have succeeded in producing pectin or alginate biconcave microparticles via droplet-based microfluidic methods^70,71^, or using moulding processes (*e.g*. particle replication in non-wetting templates, PRINT) to imprint this shape in acrylate-derived hydrogel particles^62,63,72^. Using dextran as a shape modifier, biconcave inorganic Ca(OH)_2_ microcapsules were also produced which could subsequently be used as a sacrificial template for silica deposition or polyelectrolyte layer-by-layer assembly to form similarly-shaped capsules with these materials^73–75^. Biconcave microparticles of layered polyelectrolytes or silk fibroin have been produced as well, utilising as sacrificial templates calcified RBCs, biconcave poly(lactic-co-glycolic acid) or ZnO microparticles^51,76,77^.

While biconcave (as well as many other shapes of) particles can be coated with a lipid bilayer, the shaping of GUVs themselves into non-spherical structures represents a much more complex challenge. Although it is possible to produce GUVs in monodisperse populations within the size range of RBCs (*e.g*. by utilising microfluidic architectures for step emulsification^78^ or droplet splitting^19^), the formation of GUVs with a stable biconcave shape has yet to be demonstrated. Nevertheless, GUVs have been shaped into discs, cuboids or rods with microfluidic trapping, specifically modified lipids and peptides, or by harnessing macromolecular crowding effects^79–83^. These methods for creating non-spherical GUVs, however, do not allow controlled and/or permanent shape modifications, do not reproduce the RBCs’ biconcavity or require lipid compositions that are too extrinsic from those of actual RBCs. To produce a biconcave RBC-mimicking GUV that could be deployed in vivo, permanent and reproducible shaping methods need to be developed that are compatible with relevant RBC lipid compositions and GUV production techniques. This endeavour could ideally be attained through the integration of a cytoskeleton-like structure inside a GUV, which is also how nature exerts control over cell shapes.

#### Mechanics and deformability

The mechanical properties of RBCs are defined by their cytoskeleton, which is composed of membrane protein complexes linked to a 2D network of nanofilaments (called the cortex) which line the inner side of the RBCs’ membrane (Fig. 3). The cortex is made up of spectrin dimer repeats ordered in a pseudo-hexagonal lattice^57,84^, with nodes that are centred around the transmembrane protein Band 3 which accounts for up to 25% of the RBC’s total membrane protein mass. Band 3 binds filamentous actin and many more proteins through the ‘vertical interactions’ - the ‘horizontal interactions’ being those within the cortex itself. The flexibility of this lattice ensures that RBCs can endure extreme deformations without rupturing (i.e. haemolysis), followed by a quick recovery of the original shape^85^. The RBCs’ deformability and overall mechanics are determined by membrane cohesion and the overall resistance of the cytosol. Dysregulation of ATP metabolism, ion transport and membrane cohesion due to pathology or senescence, can result in a change in surface area, volume and/or deformability, which leads to the clearance of RBCs from circulation in the spleen^60^. Besides their role in transport of oxygen and carbon dioxide, RBCs have a regulatory function which is intimately connected to their cytoskeletal mechanics. Upon their passage through capillaries, for example, release of ATP and NO is triggered by sensing of low oxygen content and by mechanotransduction pathways upon the RBCs’ deformation, as such mediating vasodilation, blood pressure and tissue oxygenation^86,87^.

Hence, to mimic these RBC properties, therapeutic artificial cells should ideally be supported by a cytoskeleton-like structure providing the necessary mechanical robustness for circulation through the microvasculature and to withstand osmotic stress^88^, while possessing an adequate deformability and the ability to recover their original shape at the same time. The importance of RBC deformability was shown by Merkel et al. and Guo et al., who demonstrated that RBC-shaped hydrogel microparticles with higher deformability displayed a longer circulation half-life compared to more rigid microparticles^51,63^ (Fig. 5).

**Fig. 6:**
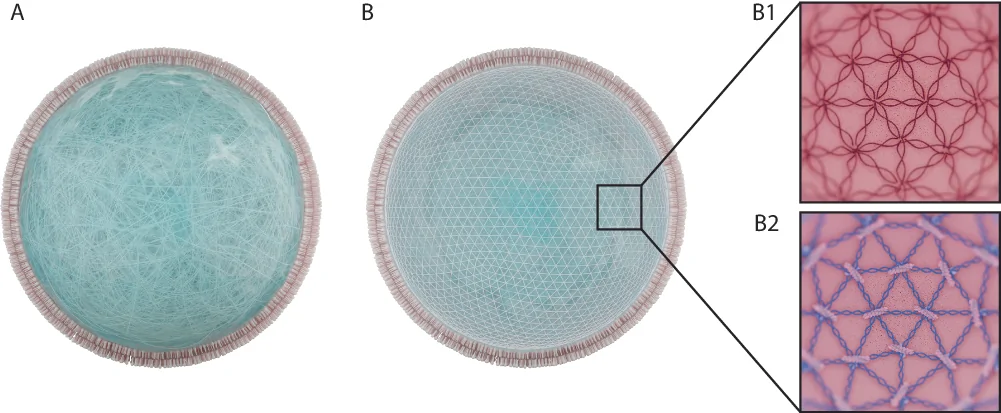
Three different routes for the formation of an artificial cytoskeleton. **A** A hydrogel-based cytoskeleton spread across the whole volume of the GUV as presented by ref. ^91^. **B** A cortex-like cytoskeleton attached to the lipid bilayer. B1: A DNA-based cytoskeleton as presented by ref. ^93^. B2: Spectrin-actin biomimetic cytoskeleton as presented by ref. ^105^.

Many attempts to encapsulate cytoskeleton-like structures in GUVs have been reported (Fig. 6). The most common strategies were based on synthetic hydrogels, since their production is relatively simple and their mechanical properties can be tuned in a variety of ways to match the physical properties of actual cells. Furthermore, they can be stimuli-responsive (*e.g*. to pH or temperature), which is useful for drug-delivery applications or mimicking physiological processes^89–91^.

Another line of research on this direction has focused on replicating the 2D architecture of cortical structures with DNA origami since its nanoscale geometries can assemble into controllable microscale structures^92^. Jahnke et al.^93^ designed DNA origami tiles that interlocked into filaments inside GUVs, resulting in the formation of a cortex-like shell that could be coupled to the membrane through DNA-cholesterol hybrids. The same group further assembled patterns of hexagonally cross-linked DNA networks inside GUVs through direct laser printing^94^. Kurokawa et al.^95^ created hexagonal DNA meshes that assembled into microspheres that, when inserted in GUVs, fused and formed a shell. Other groups created DNA skeletons with varying morphologies by varying the Mg^2+^ concentrations of the buffers^96^.

The final, most common approach to synthetically build cytoskeletal structures, has been based on the encapsulation of native cytoskeletal proteins in GUVs. More specifically, actin shells have been assembled by ion-carrier mediated Mg^2+^ influx^97–99^ or by co-formulating actin with actin-modulating proteins^100–104^. Merkle et al.^105^ reconstituted actin, spectrin and ankyrin in GUVs made from native membrane fractions, and obtained a cytoskeletal cortex that was associated to the membrane.

All these approaches, though, have produced unstructured cortical assemblies and thus far they have not been tested for their ability to support non-spherical GUVs or to enhance deformability/shape recovery in GUVs, which are all necessary features to create an RBC mimic. In addition, in order to shape GUVs in a durable and controlled way (see “Morphology and mechanics” section), the cytoskeletal structures should be anchored to the membrane, in a similar way as actin/spectrin networks are associated with Band 3 in actual RBCs. The simultaneous incorporation of cytoskeletal transmembrane proteins and cortical elements inside GUVs, however, has not frequently been reported. After all, most studies employed the IET method for the reconstitution of an actin network inside GUVs (see “GUVs” section), which is poorly compatible with the incorporation of transmembrane proteins^106^. Therefore, more straightforward approaches have traditionally been implemented by employing modified membrane-forming molecules, such as cholesterol-tagged cytoskeletal compounds, NTA-Ni-functionalised or biotinylated lipids and actin or by simply harnessing the electrostatic interactions between negatively-charged actin filaments and positively-charged lipids^29,93,103,107^.

### Membrane composition

#### Lipids

Recent research has highlighted the importance of lipid composition in RBCs as another key determinant of their function and survival. The lipid bilayer of the RBC membrane in large part consists of phosphatidylcholine (PC), phosphatidylethanolamine (PE), phosphatidylserine (PS), phosphatidylinositol (PI), and sphingomyelin (SM)^108^. While these are the main components of the RBC membrane, their ratios and distributions are subject to some natural biological variability^109^.

##### Membrane asymmetry

As in many cells, the lipid composition of the inner and outer leaflet of the membrane of RBCs is different, which is referred to as membrane asymmetry. The outer leaflet is predominantly composed of PC and SM, while the inner leaflet is mainly composed of PS and PE^108,110^. This asymmetry is important because of the physicochemical characteristics of individual lipids, *e.g*. PE increases membrane fluidity and rigidity, while PS is an essential co-factor of several membrane-bound proteins (*e.g*. Na^+^/K^+^-ATPase, protein kinase C)^111,112^. The disruption of this asymmetry has important implications in various cellular processes, *e.g*. the presence of PS on the outer membrane leaflet of RBCs is perceived as a physical injury, which promotes blood coagulation and provides a signal for their removal by phagocytes^113^. Imbalances in this asymmetry, which can occur naturally, are associated with several pathologies (*e.g*. haemolytic anaemias)^114–118^ and RBC senescence^119–121^. It has also been shown that the RBC membranes have an intrinsic membrane potential, which is independent of the presence of ions and strictly arises from lipid asymmetry. This is important, since membrane potential governs many biological processes and might affect the manner in which an RBC-mimicking GUV would function as a drug-delivery system^122^.

Further evidence of the physiological relevance of this asymmetry is that it is regulated via specialised enzymes, called lipases and scramblases, which control lipid translocation in an ATP-dependent and -independent manner, respectively. These enzymes maintain the membrane asymmetry by ‘flipping’ the lipids from inner to outer membrane leaflet and vice versa^116,123,124^. Without the activity of these transporters the collapse of this lipid asymmetry^124^ would quickly lead to RBCs being removed from the bloodstream by neighbouring phagocytes^116^.

Due to the importance of this asymmetry in a biological system, the method to produce an RBC-mimicking GUV should allow for the formation of asymmetric lipid bilayers, with emulsion transfer or microfluidic methods being the obvious choices (Table 1). Inclusion of lipid transporters should also be considered in order to maintain the membrane asymmetry necessary for the longevity and safety of the GUVs in a circulatory system, *e.g*. excessive PE and PS on the outer leaflet of the drug-delivery GUVs could lead to vesicle fusion or even thrombosis^119–121,125,126^.

#### Membrane proteins

(Trans)membrane proteins play crucial roles in maintaining the integrity of the RBCs’ membrane as well as in providing the necessary functions to fulfil their physiological roles, *e.g*. signalling, transport, adhesion, and cell-cell communication^127^. Membrane proteins are also responsible for maintaining RBCs in circulation for long periods of time by preventing their clearance through various mechanisms such as immune evasion and cell adhesion^128^.

CD47 is a transmembrane protein that is expressed on the surface of many cells, including RBCs and platelets, which interact with SIRP*α*, a receptor expressed by macrophages, acting as a ‘do not eat me’-signal^129,130^. This same mechanism can be applied to prevent the GUVs from being cleared from circulation, by integration of CD47 in the GUV phospholipid bilayer. Another membrane protein that prevents the formation of membrane attack complexes and the subsequent destruction of RBCs is CD59 which binds to the C8 and C9 proteins, thus inhibiting the formation of the complex^131^.

Another class of membrane proteins that contribute to the longevity of RBCs are glycophorins, whose sialic acid residues act as a ‘self-marker’ for the immune system, indicating that the cells are not foreign and should not be cleared^132^.

An additional advantage of integrating membrane proteins in GUVs would be to provide a biomimetic strategy to achieve targeted drug delivery by harnessing specific, cell-like ligand-receptor interactions. The production and isolation of such membrane proteins, however, still presents challenges due to the need to express them in sufficient amounts in adequate recombinant systems enabling the inclusion of complex post-translational modifications and developing effective purification and stabilisation methods. Furthermore, the incorporation of these membrane proteins into GUVs, such that their function and orientation are preserved, is still a challenging task due to the intrinsic limitations of the different GUV production strategies (as mentioned in “GUVs” section).

### Osmotic balance

Another important feature of RBCs that should be considered for production of effective therapeutic artificial cells is the RBCs’ ability to manage differences in chemical potential. The lipid bilayer membrane of cells (and GUVs) is a semipermeable barrier and, therefore, the latter are sensitive to osmotic imbalances between the internal and external environment^88,133^. The osmotic balance is directly correlated to the cellular hydration state and volume, which are properties that directly affect the RBCs’ deformability. RBCs regulate these by strictly controlling, among other parameters, intracellular Na^+^/K^+^ content, redox state, membrane surface area and haemoglobin concentration^61,134^. This control is mediated by transmembrane proteins like Na^+^/K^+^-ATPase, Band 3 and aquaporins^135,136^. In conventional GUV production protocols, osmotic imbalance is typically avoided by ensuring there is no osmolarity mismatch between the inner and outer buffers. However, to successfully deploy GUVs as RBC mimics in vivo, they should tolerate physiologically relevant levels of osmotic stress^88^. Stability against osmotic stress has already been established in GUVs by several strategies, including their decoration with a chitosan layer^137^, the inclusion of a variety of cytoskeleton structures^91,95,96^ or via magnetic aggregation^138^. Particularly, biomimetic cytoskeletal structures with larger contact area with the membrane provided superior resistance to osmotic stresses^96,133^. These studies highlight the importance of integrating support structures (i.e. a cytoskeleton) within GUVs to provide mechanical robustness against stress.

A second strategy towards this goal is to reconstitute transporter proteins in the GUVs’ membrane to allow them to regulate their own luminal environment. Provided ATP, K^+^ and Na^+^ are present in the adequate gradients, the reconstitution of Na^+^/K^+^-ATPase should permit such regulation^136,139^. Similarly, aquaporins or mechanosensitive transmembrane channels (*e.g*. such as the mammalian non-selective Piezo1 cation channels) that open in response to an osmosis-mediated influx of water or in response to mechanical stress^140^, respectively, could be incorporated in the GUV membrane. Nonetheless, the need for such advanced osmoregulation constructs is debatable, since the osmolality of blood plasma in humans usually does not vary more than 1 or 2% from the basal levels^141^. This is hypothetically endurable by plain GUVs, particularly when mechanically stabilised or when cholesterol is present in the lipid bilayer^142,143^.

### Macromolecular crowding

The interior of cells, including RBCs, is extremely crowded, with macromolecular concentrations oscillating between 50 and 400 mg/mL^144,145^. In an RBC, for example, the most abundant protein is haemoglobin with a concentration of 330 mg/mL, taking up 25% of the cytosolic volume^145^. Since other proteins are excluded from this volume, they experience a higher effective concentration which influences their folding properties, reaction rates and equilibrium constants. Compared to actual cells, the lumen of typical GUVs manufactured with the previously introduced methods is relatively devoid of macromolecules. By mimicking the inner crowded environment of RBCs in GUVs, significant stability and function gains might be achieved. It has, for example, been shown that molecular crowding results in more robust gene expression^146^ and cryopreservation^147^, and influences the assembly of a DNA origami-based cytoskeleton^93^.

Although potentially beneficial, molecularly crowded GUVs have been produced only in a limited number of studies, mainly due to the difficulty of creating GUVs in such conditions. Given the need for high encapsulation efficiencies, methods like the double emulsion techniques have mostly been used. But, as mentioned earlier, lipid bilayers produced with this method typically contain an oil pocket which disrupts membrane and protein function^148,149^. Guerzoni et al.^150^ developed a microfluidic chip for double emulsion production where first a macromolecular crowding agent (Ficoll PM70, bovine serum albumin or dextran) was encapsulated in a w/o droplet. In a second step, a w/o/w droplet was created which was later subjected to an osmotic pressure to further increase the concentration of the crowding agent. Finally the oil pocket was removed from the lipid bilayer by centrifugation. Although complete removal of the oil was not possible, the authors suggested this could be achieved using additional microfluidic techniques^151^. Despite illustrating significant steps in generating crowded GUVs, this work also highlights the limitations of current GUV production techniques to generate GUVs with biological-like features. Furthermore, the crowding agent itself is likely to have a significant impact on the final application and, therefore, the choice of such molecules needs to carefully be considered^152^.

## Alternative RBC biomimetic systems

### RBC-based systems

**Fig. 7:**
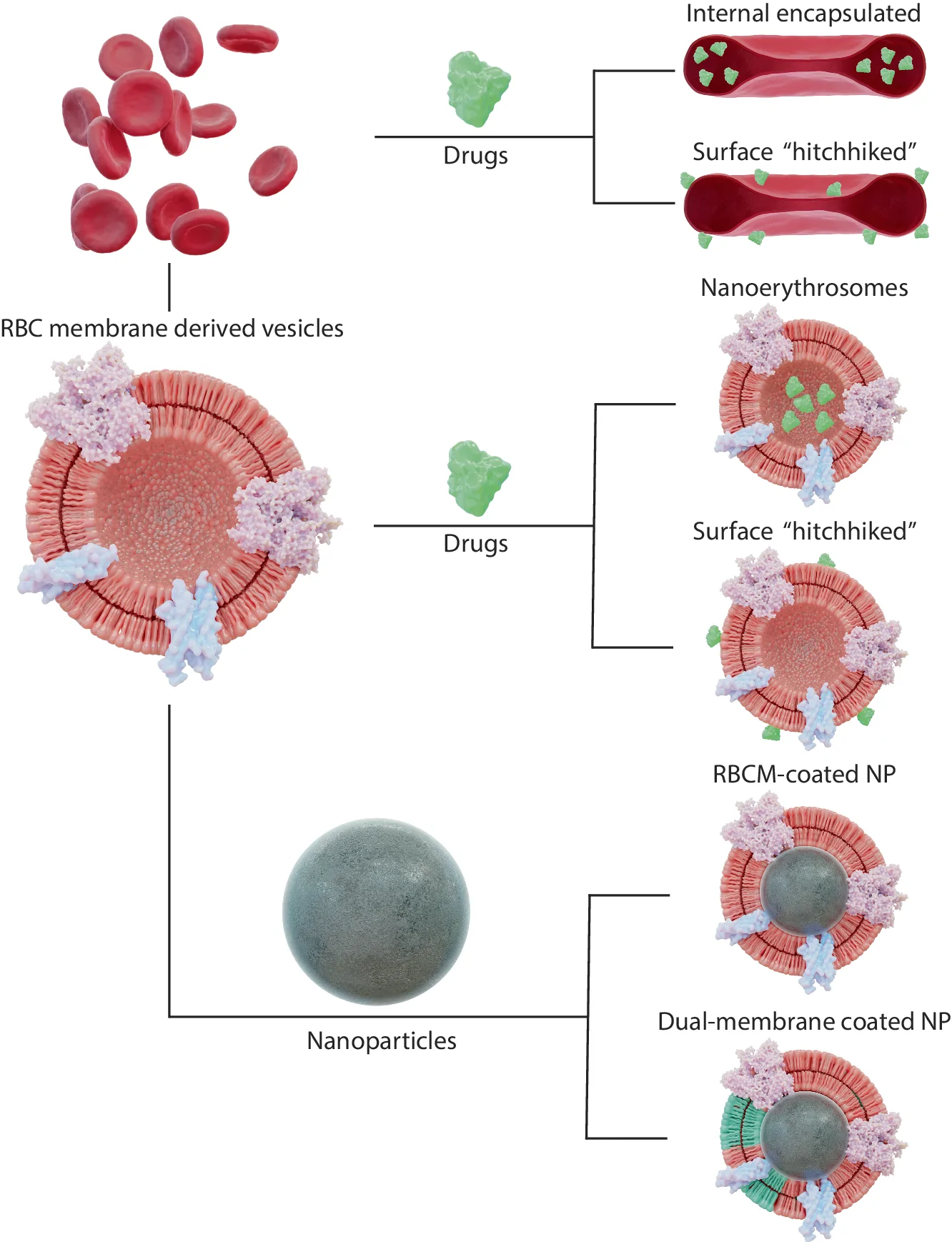
Alternatives to RBC biomimetic systems as described by ref. ^231^. RBCs can be utilised directly as a drug carrier or can be converted to smaller vesicles in/on which drugs can be attached potentially stabilised by a nanoparticle.

The use of actual RBCs modified to serve as drug-delivery systems has been demonstrated in numerous studies, with some even reaching clinical trials (Fig. 7). This approach makes use of the RBCs’ biochemical properties and consequent circulatory characteristics, which are preserved to a certain extent. Nevertheless, such systems (listed below in detail) still depend on donor-derived RBCs and are hence vulnerable to supply problems and the inherent risk of induced transfusion-associated hazards. In comparison to these, GUV-based artificial cells could be made to be safer, more universal, easier to store and would offer a larger versatility regarding encapsulation of compounds and therapeutic applications.

#### Drug-loaded carrier RBCs

With several procedures to load or coat RBCs with a drug, RBCs themselves can be used as delivery vehicles. As such, the RBCs’ innate protection against macrophage uptake and prolonged circulation time can directly be harnessed to improve drug pharmacokinetics without the need for engineering a biomimetic vehicle with similar properties. However, this strategy is not without its own issues. The ex vivo protocols to load the RBCs with the therapeutic agents are costly and might irreversibly damage the RBCs’ membrane, compromising their biocompatibility. Furthermore, there is a limit on the amount of drugs that can be encapsulated and the need to consider additional, complex strategies for controlling their release. Finally, the mechanical and rheological properties of the treated RBCs are usually detrimentally affected (although for unclear reasons), demonstrated by the fact that these systems display much shorter half-lives than their unmodified counterparts^153^ (*e.g*. ten days in mice for RBCs loaded with L-asparaginase^154^). Nonetheless, research in this field has progressed faster than with GUVs, with many RBC-based therapeutic products currently undergoing clinical trials^155–159^.

In addition to loading RBCs with drugs, these can also be deposited on the surface of RBCs. Examples include the linking of theranostics (*e.g*. nanoparticles) to RBCs via biotin-streptavidin or antigen-antibody binding, which can even be done on RBCs circulating inside the body^153,160^. This kind of ‘cellular hitchhiking’ has already been harnessed for a diversity of applications in vivo^161–164^.

Another strategy that utilises RBCs for novel therapeutic purposes, is by directly producing genetically modified RBCs from a diversity of progenitor sources (*e.g*. human hematopoietic stem cells, progenitor cells, human embryonic cells, induced pluripotent stem cells or immortalised cell lines^165^). Although the production of ‘cultured’ modified RBCs can bypass some of the disadvantages of donated RBCs (i.e. immunogenicity)^166^, this route is typically complicated by difficulties in production up-scaling, reproducibility and high manufacturing costs. Still, concepts utilising genetically modified RBCs have already been demonstrated as artificial APCs for immunotherapy applications^167^.

#### Nanoerythrosomes

Another strategy that harnesses the properties of native RBCs is embodied in the use of nanoerythrosomes (NEs), which are liposomes originating from RBCs that have been lysed to remove the original haemoglobin, and subsequently treated to form enclosed nanovesicles^168,169^. During the NEs’ formation process, their membrane can be modified and they can be loaded with drugs or fused with synthetic vesicles to create hybrid systems^170,171^. NEs are popular drug-delivery vehicles because of their increased in vivo circulation half-life and colloidal stability^172^, and they can extravasate into tumour tissue to release their cargo on the spot - in contrast to larger GUVs that would remain contained within the circulatory system^173^. Compared to GUVs, their production is relatively simple, but many of the RBC’s transmembrane proteins are lost during preparation^174^. Therapeutic formulations of NEs have already been applied in vivo with remarkably improved therapeutic results^171,175,176^.

#### RBC membrane-coated carriers

Another common strategy to enhance the targeting, delivery and circulation properties of micro- and nano-carriers is by coating them with fractions of RBC membranes. This bottom-up strategy has an enormous versatility: it is rather simple to implement and can be used with many different types of particles. Hence, it is considered by many as an important strategy for the future of drug delivery. An improved in vivo circulation half-life has been demonstrated with such systems^177–179^, even with RBC-shaped microparticles^51^. Nevertheless, and similarly to NEs, RBC-coated carriers suffer from the limitation that the extensive processing required to manufacture them results in damage of the membrane components and in changes in its composition, which detrimentally affects their stability and triggers an immune response^180^.

Finally, micro- and nano-carriers can also be coated with synthetic lipid formulations, resulting in a mechanically stabilised liposome-like carrier. For example, lipobeads (hydrogel particles coated with a lipid bilayer^181^) and coated silica microparticles have been tested in vivo with promising results^182–184^.

### Alternative materials

Vesicle-like compartments can be generated with a diversity of building blocks beyond phospholipids, including particles (forming colloidosomes)^185^, amphiphilic block-copolymers (forming polymerosomes)^186^, or mixtures of polymers and lipids^187^. According to the properties of their building blocks and the manner in which they associate, those capsules can exhibit enhanced stability and special physicochemical properties^185,188^. Albeit the functionalisation with membrane proteins is not possible in these systems, colloidosomes can display highly stable encapsulation of biomolecules in a mechanically robust shell, the particles of which can easily be modified to provide additional functions^185,189^. Amphiphilic polymers can behave similarly to fatty acids, but their molecular weights can be tuned to be up to 20-fold higher, resulting in membranes with highly adjustable physicochemical characteristics (such as permeability, viscosity and elasticity^190,191^). Since polymer membranes tend to be thicker than lipid vesicles (5–50 nm compared to 3–5 nm)^187,188^, they are typically more stable and show lower permeability^190,191^, even though incorporation of functional transmembrane proteins is challenging. Moreover, an adequate selection of the polymer blocks is necessary for in vivo applications, since many of them are not sufficiently biocompatible and biodegradable.

Lipids and polymers can also be combined to form hybrid vesicles, with lipids providing the necessary biocompatibility and functionality, while the polymers bring in additional stability and functional versatility^191^. Their manufacture, though, is complex, given the discrepancy in dimensions and/or solubilities between lipids and polymers, often resulting in a driving force towards phase separation or even vesicle disintegration^192^. KaloCyte has developed a successful example of such a hybrid system, ErythroMer™, which encapsulates haemoglobin and pH-sensitive compounds to enable pH-sensitive O_2_-release. This artificial blood substitute can additionally be freeze-dried and has shown promising in vivo results for transfusion medicine applications^193,194^.

## Beyond RBCs: adding more functionality

One of the advantages of developing RBC-like GUV carriers for in vivo applications is the possibility of integrating functionalities beyond those of normal RBCs. Different conventional biological functional units, like proteins and nucleic acids, can be incorporated in GUVs to provide a wide diversity of functions, such as a cell-free transcription and translation machinery^195^, transmembrane proteins^196^ and light production with luciferase^197^. What is more, when these biological units are exploited in non-conventional ways or when non-biological units are integrated, additional interesting functions can be integrated^198^. Examples of this include DNA origami nanopores^199^, transmembrane signal transducers built from synthetic chemicals^200–202^, magnetotactic behaviour endowed by encapsulated magnetic nanoparticles^203^ and photo-inducible release enabled by photopolymerisable lipids^204^. In the following sections, some of these extra potential functionalities are highlighted.

### Protein production

During GUV production, a cell-free protein expression system can be encapsulated to produce proteins leading to a specific biological effect, thereby potentially allowing for the spatiotemporal control of the synthesis of a therapeutic agent and its controlled release^188^. A simple example of such a system is the production of eGFP by an E. coli cell-free expression system encapsulated in GUVs^205,206^. As discussed earlier, Chen et al. (2022) produced GUVs able to express recombinant human basic fibroblast growth factor for the stimulation of pro-angiogenic activity and tissue regeneration^45^. For an additional level of control, cell-free protein production in GUVs can also be triggered by external stimuli, *e.g*. with light^188^.

### Sensing

A GUV-based RBC mimic can be potentially further modified to display additional sensing functionalities, integrating some of the complex behaviours (i.e. stimuli-responsiveness) typically displayed by actual cells. Therapeutic cell products are burdened by issues like complex genetic programmes, bio-containment and viability, while biosensing with pure cell-free expression system suffers from variable sensitivity^207^. By providing a robust outer shell, GUV-based artificial cells can mitigate the impact from external conditions while posing less of a biohazard, since they cannot replicate. Finally, the membrane can be modified with many different proteins acting as gates or receptors to improve the response selectivity. Examples of such sensing systems include detection of glucose levels in the bloodstream in vivo and sensing of histamine, Ca^2+^, bacterial acylated homoserine lactones (AHLs), hydrogen peroxide and hydrogen sulphide^7,208–211^.

### Targeted drug release

Ideally, an effective drug-delivery vehicle should release its cargo only when required and specifically at the therapeutic site of interest. By choosing the phase transition temperature of their constituent lipids, liposomes can be optimised to release their load under mild hyperthermia^212^, which can be induced locally, *e.g*. with a laser^213^. Since the pH within the tumour microenvironment is typically acidic, this property was exploited to control the delivery of drugs (*e.g*. bleomycin) from pH-sensitive liposomes^214^. These liposomes contained 2-carboxycyclohexane-1- carboxylated polyglycidol modified with distearoyl phosphatidylethanolamine, a molecule that switches from a coiled to a globular conformation upon protonation of its carboxylates, leading to the release of the drug from the liposome. External signals can also serve as a cascade-triggering signal that results in drug release, for instance GUVs with connexon nanopores whose assembly was triggered by illumination^215^. In another example, gold nanorods were coupled to the GUV surface, inducing drug release under NIR irradiation as a result of the photothermal effect^216^.

Mannose-modified liposomes were created to specifically target APCs for antigen delivery^217^. GUVs were produced from different types of special lipids which can be functionalised with different proteins: biotinylated lipids can be coupled to biotinylated proteins via streptavidin, NTA-tagged lipids can interact with His-tagged proteins and DOPE lipids can react with hydroxysuccimide-functionalised molecules. Via these methods, Staufer et al.^19^ coupled anti-CD3 antibodies and RGD-ligands to GUVs, inducing their specific interaction with a specific population of cells.

### Imaging

In addition to satisfy their intended biomedical application, the ability to assess the biodistribution of biomimetic carriers can be achieved by incorporating different types of contrast/signalling agents inside the GUVs. Magnetic nanoparticles, for example, were incorporated in biomimetic carriers to allow for their visualisation via MRI, which offered the additional benefit of enabling their magnetic manipulation^51,203^. Alternatively, fluorescently labelled particles can be incorporated for imaging purposes^218^.

### Storage

Finally, another important consideration for the therapeutic applicability of GUV-based artificial cells, is their shelf life, which is highly dependent on their production and storage conditions. With the ability to fully engineer these systems (it is, for example, possible to add lyoprotectant during the GUV production) it might be possible to store GUVs for longer periods of time than actual RBCs. The latter can only be stored for 42 days, as defined by the FDA, since they undergo a cascade of biochemical changes that adversely affect their function and properties^219,220^.

One of the most attractive approaches for long-term storage of therapeutics is lyophilisation/freeze-drying, which, for LUVs, has been shown to be effective for up to six months^221,222^. The use of lyoprotectants during lyophilisation allows bacteria and yeast cells to regain their function upon rehydration. Mammalian cells, in general, do not survive this process, but some functions still remain (*e.g*. freeze-dried platelets retain some clotting properties)^223,224^. For RBCs, the recovery rate has been shown to be less than 25% for pre-transfusion alloantibody screening^225^. These results might be improved by addition of lyoprotectants inside the cells, but this is rather difficult and the loading process might damage the cells. With GUV-based artificial cells, though, this should be less of an issue since lyoprotectants can directly be co-encapsulated during GUV production^223^.

Another option is the storing the samples in a frozen state. In the presence of glycerol, RBCs can be stored frozen for up to 10 years^226,227^. However, due to the high costs involved in the storage and removal of the glycerol and a limited shelf life after thawing, this approach has found limited practical use^227^. Furthermore, storage of extracellular vesicles and LUVs by cryopreservation has been demonstrated^228–230^, which provides a route for exploring the long-term storage of GUV-based artificial cells, which has not been done to date.

## Conclusion

In this review, the potential of GUV-based artificial cells for in vivo therapeutic use has been critically assessed. Over recent years, great progress has been made in the field of artificial cells, where GUVs featuring a wide variety of functionalities have been developed. However, only a limited number of in vivo applications with these systems has been demonstrated, and no studies have yet reported their viability to circulate in the bloodstream. Amidst numerous strategies that have been explored to enhance the circulation half-life of drug-delivery systems (which include a diversity of surface modifications and variations in vehicle morphology and mechanical properties^65^), biomimicry of RBCs represents a promising strategy that can help create a robust, durable and versatile vehicle, especially for GUVs. To this end, replication of the main features that provide RBCs with such properties via biomimicry offers the largest potential. These have been identified as size and shape, cytoskeleton, deformability, membrane composition, transmembrane proteins, osmotic balance and macromolecular crowding. Although some of these aspects can be reproduced to a satisfactory extent, the combination of several of them is still challenging and some of them (i.e. biconcavity) remain to be demonstrated. This is due to inherent limitations in the currently available techniques for GUV production, which can only accommodate the recreation of certain of these specific features separately. Therefore, for the future production of therapeutic artificial cells, it is necessary to continue to develop new GUV-manufacturing techniques enabling the creation of lipid-asymmetric vesicles that incorporate a wide set of transmembrane proteins assembled into nanodomains. At the same time these techniques would still need to allow the encapsulation of active compounds like haemoglobin (for artificial red blood cells) as well as the reconstruction of a cytoskeletal architecture providing mechanical stability and deformability, all in a molecularly crowded environment. Finally, these strategies should also be developed taking into account production scalability and cost.

Compared to RBC-based systems, the biggest advantages of GUV-based artificial cells as a delivery vehicle would be their engineerability and manufacturability: RBC-like GUVs built from the bottom up, could be manufactured with fully controlled compositions and functionalities. In contrast with liposomes, the GUVs’ size would enable the encapsulation of complex systems, such as large biological constructs and drug quantities.

A very basic, yet relevant example of a potential application of these GUV-based systems would be the ability to generate an artificial RBC mimic. In such system the presence of a biological membrane containing transmembrane proteins could provide more functions than the mere encapsulation of haemoglobin, such as the on-demand export of ATP and NO regulation, which are currently lacking in many RBC mimics. In more developed, complex systems, advanced functionalities could be integrated to transcend the normal function of an RBC. Examples of functions that could be integrated are: protein production (*e.g*. a drug), environmental sensing, stimuli-responsiveness and inclusion of lyoprotectants for improved cryopreservation. Potential applications are not limited to an advanced RBC mimic: artificial cell-like systems could be imagined that take over other types of functions like those of T-Cells for cancer treatment, for maintaining blood-sugar levels and advanced biosensors for monitoring drug levels.

Overall, the generation of in vivo-circulating, multifunctional GUV-based artificial cells has the potential to revolutionise the field of drug delivery, by providing a cell-like platform with cell-like properties, but more research is required to achieve these goals.
